# The Relationship between Multiple Substance Use, Perceived Academic Achievements, and Selected Socio-Demographic Factors in a Polish Adolescent Sample

**DOI:** 10.3390/ijerph13121264

**Published:** 2016-12-21

**Authors:** Joanna Mazur, Izabela Tabak, Anna Dzielska, Krzysztof Wąż, Anna Oblacińska

**Affiliations:** 1 Department of Child and Adolescent Health, Institute of Mother and Child, 01-211 Warsaw, Poland; joanna.mazur@imid.med.pl (J.M.); izabela.tabak@imid.med.pl (I.T.); anna.oblacinska@imid.med.pl (A.O.); 2 Department of Sexology, Counseling and Rehabilitation University of Zielona Góra, 65-729 Zielona Góra, Poland; wazkrzysztof@gmail.com

**Keywords:** perceived academic achievement, adolescence, multiple substance use, socioeconomic determinants, urban-rural differences

## Abstract

Predictors of high-risk patterns of substance use are often analysed in relation to demographic and school-related factors. The interaction between these factors and the additional impact of family wealth are still new areas of research. The aim of this study was to find determinants of the most common patterns of psychoactive substance use in mid-adolescence, compared to non-users. A sample of 1202 Polish students (46.1% boys, mean age of 15.6 years) was surveyed in 2013/2014. Four patterns of psychoactive substance use were defined using cluster analysis: non-users—71.9%, mainly tobacco and alcohol users—13.7%, high alcohol and cannabis users—7.2%, poly-users—7.2%. The final model contained the main effects of gender and age, and one three-way (perceived academic achievement × gender × family affluence) interaction. Girls with poor perception of school performance (as compared to girls with better achievements) were at significantly higher risk of being poly-users, in both less and more affluent families (adjusted odds ratio (OR) = 5.55 and OR = 3.60, respectively). The impact of family affluence was revealed only in interaction with other factors. Patterns of substance use in mid-adolescence are strongly related to perceived academic achievements, and these interact with selected socio-demographic factors.

## 1. Introduction

Despite a decreasing trend, the widespread use of psychoactive substances by young people continues. Alcohol is the most frequently used substance by adolescents. Slightly fewer adolescents smoke tobacco, and even fewer reach for illegal drugs, the list of which is constantly being modified [1]. The use of psychoactive substances increases with age, and taking more than one is a frequent phenomenon [2,3,4]. Research interest is focused on adolescents at the age of tobacco, alcohol, and drug initiation (usually at 13 years of age) and in subsequent years, when the first symptoms of problems connected with abuse begin to appear [5,6]. Longitudinal studies from early adolescence to early adulthood enable the prediction of problem behaviours [7] and indicate the need for intervention during the critical period, taking into account various social groups. Attention should be paid to multiple substance use and thereby more complex “user profiles” as well as their social and cultural background. The impact of socioeconomic factors on psychoactive substance use by young people is studied at various levels, taking into consideration the socioeconomic status of the family [8], the social structure of students in a given school [9], neighbourhood features [10], and the country’s macroeconomic indicators [11].

The expected negative correlation between substance use and family socioeconomic status has been confirmed in adult studies [12]. According to the literature review conducted by Hanson and Chen [13], such negative correlation was most frequently described in relation to tobacco smoking (30 out of 44 studies). In case of alcohol and cannabis, the negative correlation was found in 8 of 28, and 4 of 25 studies, respectively. Various results indicate that there is a need to look for other determinants associated with material factors, taking into account cultural and social differences in the studied country.

Results from the HBSC (Health Behaviour in School-aged Children) study suggest the existence of regional cultural determinants in Poland. The lowest rates of psychoactive substance use are recorded in the south-eastern part of the country, with the lowest urbanization rate, relatively lower level of economic development, but a significant attachment to traditional values [14]. Hełpa-Liszowska points to a number of factors which may cause a deepening of social differences between metropolitan and rural areas; some of these being directly or indirectly related to education. These factors include: failure to adjust professional qualifications to the current needs of the job market (which results in unemployment), a lower standard of living, limited access to information and opportunities to enhance knowledge, as well as often lower aspirations in life [15]. A lower level of parent education is a predictor of lower educational aspirations of adolescent children and, as a consequence, a lower level of education in adult life. In addition, during the last few years, there has been a national discussion about cultural changes in Poland’s rural areas, which may affect the way young people live. The stereotypical perception of traditional, conservative rural communities being less susceptible to new civilisation patterns than metropolitan communities gradually vanishes. The fact that adolescents in rural areas reach for psychoactive substances may be perceived as their way of dealing with day-to-day boredom and an inferiority complex with regard to their peers from urban areas. Moreover, the mechanisms of social (and parental) control, typical for smaller communities, attached to traditional values, have become less effective. Thus, the need to raise education levels in rural areas becomes even more significant.

The association between academic achievement and risk behaviours among young people requires more in-depth analysis. Depending on whether poor achievements are considered a cause or an effect of the use of psychoactive substances, we speak about social causation or social selection [16]. According to social causation hypothesis, living in worse conditions increases the risk of negative health outcomes or health compromising behaviour. The social selection hypothesis suggests that some negative health outcomes lead people to drift into the lower social class or to never escape poverty. 

Poland is an interesting country when educational and social inequalities and their impact on health indicators are considered. In the light of the results of two successive series of HBSC studies (2009/2010 and 2013/2014) Poland led the ranking of countries in terms of the difference between rich and poor families with regard to the percentage of students achieving very good school results [17]. It may therefore be concluded that academic achievements represent the hidden social variable. They are strongly correlated with the target level of education, which is already a generally recognized indicator of social position. Inhabitants of small cities may be a privileged group since they are to a lesser degree affected by problems typical of large urban agglomerations as well as by problems seen in rural areas [18]. Many authors abandon traditional comparisons between cities and rural areas in order to consider a broader social context, which takes lifestyle and social norms into account. Dutch studies concerning the conditions of sustained regional development quote a classification of several dozen indicators of ecological, socio-cultural, and economic capital [19]. It should also be noted that health indicators, including risk behaviours, also constitute a part of local socio-cultural capital. 

To our best knowledge, there are no studies that combine the abovementioned issues and present an assessment of the impact of academic achievements on risk behaviours in mid-adolescence, taking into account both demographic and social factors. 

The aim of the study was to establish the prevalence and selected socio-cultural determinants of the patterns of psychoactive substance use among lower secondary school students. The following research questions were formulated.

- What patterns of psychoactive substance use by adolescents may be currently distinguished in Poland?
- Are young people who consider themselves to do better at school than their peers less prone to co-occurrence of risk behaviours measured in terms of multiple substance use?
- To what extent do gender and economic status of the family affect the adoption of a given pattern of psychoactive substance use and how does it correspond with academic achievements?
- Do features of the place of living (level of urbanization, deprivation) modify the studied correlations?

A hypothesis was adopted that the correlation between use of psychoactive substances and academic achievements is modified by socio-demographic factors such as gender, place of living, and family affluence.

## 2. Materials and Methods

### 2.1. Sample and Procedure 

Data were derived from the latest round of the HBSC survey, which is performed periodically every four years for three age groups, 11, 13, and 15 years of age. In the current study, filling in a questionnaire for the oldest age group, without missing data about the use of all three psychoactive substances (tobacco, alcohol, cannabis), was a criterion for inclusion in the analysis. In total, 1202 persons (including 46.1% boys and 53.9% girls) were qualified for the analysis. The average age was 15.64 years (SD = 0.31). The vast majority (86.9%) were in the third year of lower-secondary school, and the remaining 13.1% were in the first year of various types of upper-secondary schools (both general and vocational). The individual schools were selected from the list of all secondary schools in Poland provided by the Ministry of National Education. The multi-stage cluster sampling was applied, where school classes was the main sampling unit. The student response rate was estimated to be 86.1%. It was a representative national sample embracing students from 93 schools in all 16 main provinces. In most cases one class in each school was surveyed, and students who fulfilled the age criterion were qualified for analyses in accordance with the HBSC protocol.

### 2.2. Variables and Indicators 

Questions about selected psychoactive substance use were adapted to the HBSC protocol from the ESPAD (European School Survey Project on Alcohol and Other Drugs) questionnaire. Among other questions, young people were asked about the number of days during the last month when they smoked tobacco, drank alcohol, and smoked cannabis (seven response categories from never to 30 days or more). After encoding responses into three categories of frequency the answers provided the basis for defining patterns of using psychoactive substances (the k-means method). Four patterns were defined empirically: persons avoiding psychoactive substances (non-users); persons smoking tobacco and drinking alcohol but not using cannabis (mainly tobacco and alcohol); persons using alcohol frequently and cannabis from time to time but avoiding tobacco (high alcohol); and those who frequently used all three substances over the last 30 days (poly-users). An alternative solution with five clusters was rejected since very small numbers were obtained in two of the clusters. The characteristics of the clusters are presented in Table 1.

The newest version of the Family Affluence Scale (FAS) was applied [20,21,22], and a standardized FAS z-score was calculated, based on the principal components method, after stratification according to the place of living. It had a normal distribution (*p* = 0.357; Kolmogorov-Smirnov test).

Three categories of localities were taken into account: large cities (more than 100,000 inhabitants), small towns, and rural areas. In addition, three questions were asked concerning the level of deprivation in the neighbourhood (LAP scale—local area perception), also derived from the HBSC protocol. Individual items of scale related to the sanitary condition of the surroundings, the technical condition of buildings, and the presence of groups of young people causing problems. A standardized index was obtained using the same method as for the FAS scale. Both FAS and LAP were recoded into three categories, where the first represents about 20% of the sample living in the most disadvantaged conditions.

Academic achievements were assessed on the basis of the students’ subjective evaluation, defining the perceived academic achievement measure. Students marked how their achievements are evaluated by teacher(s) in comparison with other students in the class. The question was validated in some HBSC countries [23] by comparing it with more objective indicators of school performance. For the purpose of various analyses four categories of answers were limited to three: below average or average, good, and very good.

### 2.3. Statistical Analysis

Behavioural patterns were characterized in terms of use of tobacco, alcohol, and cannabis, presenting frequency according to the original full score, which was not previously the base for clustering. The frequency of occurrence of individual patterns was compared depending on the features of the neighbourhood, perceived school achievement, and family affluence (chi-squared test). In a multivariate analysis, multinomial logistic regression models were estimated with clusters of substance use as a dependent variable and cluster one (non-users) as the reference category. A model that recognized only the main effects was estimated, and then all possible interactions were examined, including the second, third, and fourth levels. As goodness-of-fit statistic, the pseudo R-squared Nagelkerke coefficient was applied. 

The analyses were carried out within the framework of a project integrated with HBSC studies, financed by the National Science Centre (2013/09/B/HS6/03438). The SPSS v.17 statistical package was used. 

The Bioethical Committee of Institute of Mother and Child in Warsaw, Poland approved the study protocol signed on 28 January 2015 (decision no 1/2015). The opinion of the Committee included the content of the questionnaire, the study schedule, as well as the procedure for obtaining the consent of parents and students.

## 3. Results

### 3.1. Patterns of Psychoactive Substance Use

In the studied group 23.0% of respondents had smoked tobacco, 40.8% had drunk alcohol, and 10.2% had smoked cannabis within the last 30 days. The strongest differences associated with gender were found with regard to cannabis smoking: 12.5% of boys had used cannabis and 8.3% of girls (*p* = 0.016) on at least one of the last 30 days. Tobacco smoking on at least one day was reported by 20.5% of boys and 25.2% of girls (*p* = 0.055). Alcohol had been consumed on at least one day by 40.1% of boys and 41.5% of girls (*p* = 0.631).

The distribution of the four patterns of psychoactive substance use in the whole sample is presented in Table 1. Nearly 72% of respondents were defined as non-users. Persons from the fourth cluster, who frequently used all three substances (7.2%), are recognized as a special increased risk group. Cluster four adolescents used tobacco and cannabis more frequently than peers from clusters two and three, while the frequency of alcohol drinking was the highest in cluster three. The average number of substances was significantly lower in cluster three than in cluster two. The agreement between the division into clusters based on the frequency of use and the number of substances was high (κ = 0.576; *p* < 0.001). 

### 3.2. Determinants of Psychoactive Substance Use and Selected Interactions

The structure of the population according to the presented patterns differed by gender (Table 2). Girls more frequently than boys combine tobacco smoking with moderate alcohol consumption (cluster two), while boys more frequently than girls qualified to the group combining more intensive alcohol drinking with cannabis smoking (cluster three). A clearly worse distribution of behavioural patterns was observed among young people with poor perception of academic achievement. 

Statistically significant differences were not recorded for any of these, although in the case of the LAP scale a tendency was noted (*p* = 0.073). When the place of living is considered the best distribution of patterns appears in rural areas, in contrast to small towns. 

An association between socio-demographic variables was also noted. A strong association between perceived school performance and family affluence was confirmed. In the subsequent FAS groups the percentage of students who perceived their achievements as very good was 9.5% in low, 13.4% in medium and 20.1% in high FAS category (*p* = 0.002). The FAS and LAP scales correlated weakly, although significantly, with each other (*r* = 0.140; *p* < 0.001).

**Figure 1:**
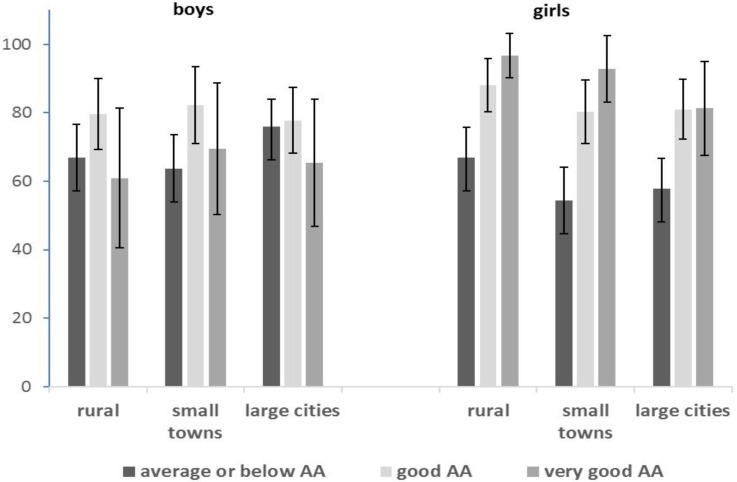
Non-users cluster (% with 95% confidence interval) by gender, perceived academic achievement (AA) and residence location.

A linear increase in the percentage of non-users combined with an improvement in the perception of academic achievements was only observed among girls (Figure 1). The respective percentages of girls in the first cluster were higher in rural areas than in cities. Among boys the protective effect of better perception of academic achievement was weaker than among girls and the strongest in small towns (non-linear correlation). The percentage of boys in the non-users group was even lower in the group of students who perceived their achievement as very good in comparison with the middle group. 

### 3.3. Multivariate Analysis 

A series of multinomial regression models was estimated, where four patterns of substance use were the dependent variable. The models enable a comparison of the effect of the analysed factors dependent on which behavioural pattern is compared with the reference category (non-users). In the model containing only the main effects, three variables proved to be significant: gender, age, and perceived academic achievement (Table 3). 

Only perceived poor academic achievement increased the risk of any substance use compared to non-users. Gender has an impact (to the disadvantage of boys) on heavy alcohol drinking (cluster three), while increasing age has an impact on tobacco smoking with moderate alcohol consumption (cluster two). Moreover, residence location and LAP were significant predictors for the cluster of poly-users at the level of *p* = 0.073 and *p* = 0.093, respectively. Some rejected variables show an interaction with academic achievement, which is additionally modified by gender. Thus, another model was proposed recognizing the effects of interaction, instead of the main effect of perceived academic achievements. Comparing those two models, the value of pseudo R-squared Nagelkerke increased from 0.078 to 0.101. The impact of poor academic achievement according to gender and FAS subgroups is presented in Table 4. The OR indicators illustrate the risk of being in the respective clusters of youth using psychoactive substances associated with poor academic achievements. In addition, by means of the interaction effect, we can control the impact of other factors. OR indicators are analysed in four groups distinguished in terms of gender and family affluence. The effect of academic achievements on the use of psychoactive substances is only visible among girls. Girls with poor perception of school performance (as compared to girls with better achievements) are at a significantly higher risk of being poly-users, both in less and more affluent families. In the relatively wealthier families the risk of finding oneself in cluster two also increased significantly.

A series of alternative multinomial logistic regression models with the same outcome measure could be proposed. For example, a significant interaction between residence location, gender, and perceived academic achievement was found. Girls with poor school performance were at a higher risk of being in the cluster of poly-users as compared to those with better perceived academic achievements only in large cities (OR = 3.36; 95% CI: 1.14–9.87; *R*-squared = 0.111).

## 4. Discussion

This study is based on data collected in the 2013/2014 school year among 1202 Polish teenagers. Potential socio-demographic determinants of various patterns of psychoactive substance use were noted, assuming the subjective measure of school performance as the main factor. Over 70% of respondents were found to be in the most favourable pattern (no use or sporadic use). No cluster with single substance use was established. A division into four behavioural patterns appears in studies by other authors, with a similar share of extreme categories. However, in American studies of young people of a similar age [24], persons described as predominantly cannabis users were identified; this is a group which is scarcely represented in Poland. 

The analyses are based on the results of the HBSC study, which, similarly to ESPAD, is often used for monitoring the frequency of psychoactive substance use by adolescents in many countries. Many studies point to a declining trend in use in various geographical and cultural regions [25,26,27]. A comparison of the rankings of countries published in successive international HBSC study reports has a unique comparative importance. Despite the decreasing frequency of tobacco smoking, Poland occupies a gradually worse position in these rankings. Even more importantly, Poland has moved to the group of countries with the highest rate of cannabis smoking [20]. It seems that traditional comparisons of the frequency of psychoactive substance use ought to be supplemented with analyses of changes in behavioural patterns, taking into account gender and social groups.

A simple analysis of contingency tables did not confirm any effect of residence location, family affluence, or neighbourhood deprivation on the patterns of psychoactive substance use. Interesting correlations were revealed only in the analysis of interactions with perceived academic achievements, which indicates that different mechanisms of health-affecting risk factors may be revealed in different populations. In an assessment of the results obtained with reference to the research questions raised, three aspects of the analysis must be noted: (1) the influence of residence location as a socio-cultural factor unique for national statistics; (2) the dominating impact of subjective academic achievements and their interaction with other factors; (3) the limited effect of family affluence, which becomes apparent only in interaction with other variables.

### 4.1. Residence Location

In the light of the data from literature, the association between psychoactive substance use and the level of urbanization is equivocal. According to some researchers the problem applies more to urban than to rural communities [28,29]. Conversely, there are examples of studies which confirm more widespread use of these substances in rural areas [30]. 

Similar to the previous Polish studies, our analysis indicated that for girls the urban community is a risk factor contributing to youth lifestyle and behaviour, while the rural community is a risk factor for boys. This is probably the result of not only living conditions, but also of cultural differences. In this part of Europe in rural areas people tend to be more attached to traditional masculine and feminine role models and social bonds are stronger. According to the latest report from a Study of Incomes and Living Conditions (EU-SILC) of the Polish population [31], a higher level of social trust and a stronger feeling of security is an advantage of rural areas, while limited access to cultural and educational services, trade facilities, and public transport are disadvantages. It is difficult to unequivocally conclude which of the residence locations is better from the point of view of the development and education of young people. The constantly increasing number of families enjoying the privileges of both urban as well as rural areas, without being indigenous inhabitants of the latter, is also difficult to capture. 

It is very likely that residence location affects the process of disappearance of differences between boys and girls with respect to the frequency of taking up risk behaviours also for cultural reasons. Internal factors of this type, unique for a given country, ought to be taken into account in studies concerning behaviour convergence. The tradition of more than 25 years of HBSC studies in Poland points to various phases of this process. With regard to tobacco smoking, at first there were differences to the disadvantage of boys regardless of the place of residence, which began to disappear more quickly in cities [32]. 

### 4.2. Perceived Academic Achievement in Relation to Family Affluence

Our study results confirmed that perceived academic achievements are strongly determined by family affluence, while affluence status does not directly affect the patterns of use of psychoactive substances. Analyses concerning mediators of the correlations between use of psychoactive substances and socioeconomic status or school performance have already been conducted. Andrews and Dunkan [33] indicated the mediatory role of the feeling of personal efficacy and family relations on the association between academic achievements and psychoactive substance use. Barr [34] pointed to health problems in the family as a mediator of the relationship between affluence and academic achievements. Sznitman et al. [35], analysing macroeconomic indicators, confirmed the mediatory role of emotional welfare. Lynskey and Hall [36] conducted a review of studies concerning the correlation between cannabis use and school performance and concluded that lifestyle and social context, including membership in deviant groups, leaving school, and the intensity of other risk behaviours, are the most likely mediators. 

Family affluence as an isolated factor does not affect the outcome measure, but interacts with other factors. The expected drop in frequency of psychoactive substance use in rich families was observed among young people with better perception of academic achievements. In groups with poor school performance, a high level of affluence becomes an additional risk factor. The mechanism of association between high family affluence and good academic achievements is in itself frequently discussed in literature and may be explained by reference to better access to teaching aids and additional extracurricular activities in richer families, more effective upbringing by families with higher social capital, better family communication, and better health of the children and parents [34,37]. 

### 4.3. Strenghts and Weaknesses of the Study

The constraints of the study result from simplified definitions of independent variables, including residence location and academic achievement. Reservations may also be raised regarding the application of the simplest statistical method of identifying patterns of substances use.

Some developed countries have chosen to introduce scales for measuring urbanisation related to post codes, such as Beale’s scale. In Poland, only 30 cities have a population exceeding 100,000, which makes it difficult to expand the category of big metropolitan areas. The status of “city” or “village”, often having historical roots, does not seem to be the only important issue here; the entire structure of the local residential network also matters. Attention is drawn to the relationship between adjacent cities and villages, their distances, and the role which bigger cities play in integrating the population of the region.

Another constraint is caused by limiting the study to only three psychoactive substances and the small number of factors taken into account, potentially affecting the distribution of behavioural patterns. However, results of this type have a specific cognitive aspect, indicating basic socioeconomic determinants which ought to be taken into consideration in more complex analyses. 

The sampling method can be considered as another limitation of the study. Small numbers of students were surveyed in any single school and in each of the administrative regions. However, the risk of the use of correlated data has been minimized by considerable geographical variation and by the large number of surveyed schools.

Despite the abovementioned methodological constraints, the study has many strong points. Socioeconomic variables were built or standardized in a way that measures the relative position of respondents in relation to others living or studying in similar conditions. Another advantage of the study is the application of multinomial regression as an original analytical approach. Classical methods of linear and binomial logistic regression do not enable the modelling of categorized dependent variables. Studies using multinomial regression, or a similar method of ordinal regression, appear sporadically in the literature related to the use of psychoactive substances [38]. 

### 4.4. Practical Implications

The results of studies devoted to the association between perceived academic achievement and intensity of risk behaviour quite clearly indicate a negative correlation [39,40,41,42]. Many authors have also pointed that this may be a mutual correlation [43,44]. Students with high academic achievement are usually persons for whom education has a high position in the hierarchy of values. Those who engage in risk behaviours have worse perception of results at school, and this is associated with a diminished motivation to study, higher stress, involvement in deviant peer groups, deteriorating family relations [45], or the physical effects of using psychoactive substances [33]. The added value of this study is that it points to the fact that perceived academic achievement strongly interacts with other factors and has a stronger protective effect for girls than for boys. 

Translating the results obtained into practice in public health and school health promotion, it is worthwhile to refer to researchers who recommend acknowledgement of the bilateral association between academic achievement and risk behaviour. According to Crosnoe, we deal with two important behaviours in positive development, which may act as risk factors for one another [46]. Prevention programs ought to interrupt the circle of negative connections. Firstly, activities should be aimed towards supporting protective factors, particularly the positive influence and support of the family and peers, who constitute a sort of buffer for adolescents with poor results at school. Another important factor is the feeling of belonging to the school and respect for school as an institution. It is also advisable to encourage the motivation to study and participate in extracurricular activities, which may provide a source of feelings of success [47]. Thirdly, better understanding of the reasons and mechanisms of co-occurrence of problem behaviours should form the basis of prevention programs. 

In the light of our study the level of family affluence is another factor which can affect positive development of adolescents, while gender additionally modifies the studied correlations. From the perspective of the country’s needs it is particularly important to implement prevention programs directed at risk behaviours among girls, where less favourable trends are being observed. In programs of this type, it is recommended to take into account the specific features of the urban and rural environment and various social groups. Another issue to be noted is support for programs providing equal educational opportunities for young people from poor families.

## 5. Conclusions

Concluding, we can assume that regardless of socio-cultural disparities between countries, differences observed within countries may influence the frequency of psychoactive substance use and propensity to use multiple substances. As the main conclusion, it was found that patterns of substance use in mid-adolescence are strongly related to perceived academic achievements, which in turn interact with selected socio-cultural factors.

## Figures and Tables

**Table 1:** Patterns of psychoactive substances use in order to their prevalence in population.

Cluster Description	*n*	%	Mean No. of Substances	Mean No. of Days in Last 30 Days *		
				Tobacco	Alcohol	Cannabis
1. Non-users	864	71.9	0.26 ± 0.44	0.04 ± 0.24	0.34 ± 0.63	0.05 ± 1.05
2. Mainly tobacco and alcohol	165	13.7	1.82 ± 0.39	13.94 ± 11.59	4.11 ± 5.89	0.00 ± 0.00
3. High alcohol and cannabis	87	7.2	1.36 ± 0.55	0.05 ± 0.28	9.26 ± 8.82	3.16 ± 8.23
4. Poly-users	86	7.2	2.88 ± 0.32	20.12 ± 10.93	7.54 ± 9.00	8.98 ± 10.40
Total	1202	100.0	0.74 ± 0.93	3.39 ± 8.43	2.02 ± 4.99	0.91 ± 4.36

* Days of substance use estimated on the basis of seven original response categories.

**Table 2:** Socio-demographic characteristics of the clusters * (%).

Independent Variable	*n*	Cluster 1	Cluster 2	Cluster 3	Cluster 4	*p* Value
Gender						
Boys	551	71.8	10.0	10.0	8.2	<0.001
Girls	651	71.9	16.9	4.9	6.3	
Grade						
9th	1045	72.9	12.7	7.0	7.4	0.04
10th	157	65.0	20.4	8.9	5.7	
Perceived academic achievement (AA)						
Average or below	623	64.2	18.9	8.2	8.7	
Good	393	81.4	7.4	6.9	4.3	<0.001
Very good	162	79.1	8.0	4.9	8.0	
Residence location						
Rural areas	390	74.3	11.3	8.2	6.2	
Small towns	367	68.7	16.9	6.5	7.9	0.32
Large cities	445	72.4	13.2	7.0	7.4	
Family affluence (FAS)						
Low	200	72.0	12.5	6.5	9.0	
Average	743	72.9	13.3	6.5	7.3	0.37
High	200	70.0	14.5	10.5	5.0	
Local areas perception (LAP)						
Low	194	66.5	14.4	9.3	9.8	
Average	714	73.7	13.6	6.0	6.7	0.07
High	187	72.2	11.8	11.2	4.8	

* Description of clusters as in Table 1.

**Table 3:** Multivariable estimates from the final multinomial logistic regression * with only main effects.

Main Effect of	Referent Group	Cluster 2 Mainly Tobacco and Alcohol			Cluster 3 High Alcohol and Cannabis			Cluster 4 Poly-Users		
		OR	95% CI		OR	95% CI		OR	95% CI	
Gender (boys)	Girls	0.67	0.46	0.98	**2.13**	**1.32**	**3.43**	1.31	0.81	2.13
Grade (10th)	9th	**1.84**	**1.15**	**2.96**	1.45	0.75	2.80	1.03	0.49	2.16
Poor perceived academic achievements	at least good	**2.86**	**1.93**	**4.23**	**1.62**	**1.01**	**2.60**	**1.90**	**1.16**	**3.12**
Rural resident	urban	0.75	0.50	1.12	1.10	0.68	1.80	0.60	0.34	1.05
Low family affluence	average or high	0.90	0.56	1.45	0.84	0.45	1.58	1.19	0.66	2.14
Low local area status	average or high	1.20	0.75	1.91	1.48	0.84	2.61	1.64	0.92	2.90

* Reference category Cluster 1—non-users; results significant at *p* < 0.05 are bolded; OR—adjusted odds ratio; CI—confidence interval.

**Table 4:** Interaction effect estimates from the alternative multinomial logistic regression * with main effects (age, gender) and three-way interaction (perceived academic achievement × gender × FAS).

Gender and Family Affluence (FAS) Level in the Interaction	Cluster 2 Mainly Tobacco and Alcohol			Cluster 3 High Alcohol and Cannabis			Cluster 4 Poly-Users		
	OR	95% CI		OR	95% CI		OR	95% CI	
Boys × Low FAS	2.06	0.82	5.21	1.47	0.61	3.56	0.59	0.13	2.73
Boys × Average or high FAS	1.61	0.81	3.20	1.06	0.56	2.01	1.72	0.83	3.53
Girls × Low FAS	2.41	0.98	5.95	2.19	0.66	7.35	**5.55**	**1.97**	**15.66**
Girls × Average or high FAS	**5.22**	**2.99**	**9.13**	2.17	0.96	4.91	**3.60**	**1.55**	**8.33**

* FAS—family affluence; reference category Cluster 1—non-users; results significant at *p* < 0.05 are bolded; OR—adjusted odds ratio; CI—confidence interval; OR associated with poor perception of school achievements.
